# Transcriptome analyses of the cortex and white matter of focal cortical dysplasia type II: Insights into pathophysiology and tissue characterization

**DOI:** 10.3389/fneur.2023.1023950

**Published:** 2023-03-15

**Authors:** Guilherme Rossi Assis-Mendonça, Maria Carolina Pedro Athié, João Vitor Gerdulli Tamanini, Arethusa de Souza, Gabriel Gerardini Zanetti, Patrícia Aline Oliveira Ribeiro de Aguiar Araújo, Enrico Ghizoni, Helder Tedeschi, Marina Koutsodontis Machado Alvim, Vanessa Simão de Almeida, Welliton de Souza, Roland Coras, Clarissa Lin Yasuda, Ingmar Blümcke, André Schwambach Vieira, Fernando Cendes, Iscia Lopes-Cendes, Fabio Rogerio

**Affiliations:** ^1^Department of Pathology, School of Medical Sciences, University of Campinas (UNICAMP), Campinas, SP, Brazil; ^2^The Brazilian Institute of Neuroscience and Neurotechnology (BRAINN), Campinas, SP, Brazil; ^3^Department of Translational Medicine, School of Medical Sciences, University of Campinas (UNICAMP), Campinas, SP, Brazil; ^4^Department of Structural and Functional Biology, Institute of Biology, University of Campinas (UNICAMP), Campinas, SP, Brazil; ^5^Department of Neurology, School of Medical Sciences, University of Campinas (UNICAMP), Campinas, SP, Brazil; ^6^Department of Neuropathology, University Hospital Erlangen, Erlangen, Germany

**Keywords:** focal cortical dysplasia, transcriptome, cholesterol biosynthesis enzymes, humanin-like 12, GPNMB, molecular biomarker, immunohistochemistry

## Abstract

**Introduction:**

Focal cortical dysplasia (FCD) is a common cause of pharmacoresistant epilepsy. According to the 2022 International League Against Epilepsy classification, FCD type II is characterized by dysmorphic neurons (IIa and IIb) and may be associated with balloon cells (IIb). We present a multicentric study to evaluate the transcriptomes of the gray and white matters of surgical FCD type II specimens. We aimed to contribute to pathophysiology and tissue characterization.

**Methods:**

We investigated FCD II (a and b) and control samples by performing RNA-sequencing followed by immunohistochemical validation employing digital analyses.

**Results:**

We found 342 and 399 transcripts differentially expressed in the gray matter of IIa and IIb lesions compared to controls, respectively. Cholesterol biosynthesis was among the main enriched cellular pathways in both IIa and IIb gray matter. Particularly, the genes *HMGCS1, HMGCR*, and *SQLE* were upregulated in both type II groups. We also found 12 differentially expressed genes when comparing transcriptomes of IIa and IIb lesions. Only 1 transcript (*MTRNR2L12*) was significantly upregulated in FCD IIa. The white matter in IIa and IIb lesions showed 2 and 24 transcripts differentially expressed, respectively, compared to controls. No enriched cellular pathways were detected. *GPNMB*, not previously described in FCD samples, was upregulated in IIb compared to IIa and control groups. Upregulations of cholesterol biosynthesis enzymes and *GPNMB* genes in FCD groups were immunohistochemically validated. Such enzymes were mainly detected in both dysmorphic and normal neurons, whereas GPNMB was observed only in balloon cells.

**Discussion:**

Overall, our study contributed to identifying cortical enrichment of cholesterol biosynthesis in FCD type II, which may correspond to a neuroprotective response to seizures. Moreover, specific analyses in either the gray or the white matter revealed upregulations of *MTRNR2L12* and GPNMB, which might be potential neuropathological biomarkers of a cortex chronically exposed to seizures and of balloon cells, respectively.

## 1. Introduction

Epilepsy is a neurological disease characterized by recurrent unprovoked seizures, which may be caused by different brain disorders. Most patients have a satisfactory clinical outcome with appropriate medications. However, seizures may be pharmacoresistant and have detrimental and even life-threatening consequences (1, 2). Around 30% of the patients with focal epilepsies are candidates for surgical treatment, and their clinical outcome depends on the pathological condition causing the seizures (3). The most common causes leading to medically intractable epilepsy are hippocampal sclerosis, long-term epilepsy-associated brain tumors, and malformation of cortical development (MCD) (4). Particularly, MCDs represent a wide range of lesions arising from the disruption of steps of cortical formation: cell proliferation, cell migration, and cortical organization. Although the pathogenesis is still unclear, specific genetic defects have been identified (5–7).

The incidence of MCDs in individuals submitted to epilepsy surgery varies among centers. However, focal cortical dysplasia (FCD) is the most frequently reported MCD. FCD is a structural lesion with different sizes, locations, and histopathological findings (7). Clinically, seizure features depend on the FCD localization, and electroencephalography mostly shows epileptiform discharges spatially correlated with the lesion. On magnetic resonance imaging (MRI), FCDs may present with cortical thickening associated with cortical–white matter junction blurring, a hyperintense signal on T2-weighted images, and an abnormal pattern of sulci and gyri (1, 4). Historically, the neuropathological classification of FCD has been as variable as its broad morphological spectrum (4, 7–9). Recently, the International League Against Epilepsy (ILAE) has reviewed and updated its neuropathological classification system for FCD. Specifically, FCD type II refers to isolated lesions characterized by dyslamination of the cortex and dysmorphic neurons without (IIa) or with balloon cells (IIb) (10, 11). It also incorporated multidimentional data, such as electroclinical, imaging and genetic findings into the classification of FCDs (12).

In this context, we designed an original high throughput study by using RNA-Seq to perform transcriptome analysis (12) and identify differentially expressed mRNAs in the cortex and white matter of individuals with epilepsy due to FCD type IIa or IIb. Even though other authors investigated FCD transcriptome (13–15), the gray and white matters were studied as homogenates, gene expression protocols were different from ours and fewer FCD cases were considered. Moreover, we compared the transcriptome profiles of the two lesions with those obtained from brain samples from autopsied control individuals without a history of neurological disease. Then, immunohistochemical validation was performed for selected molecules. Also, we aimed to identify molecular pathways in FCD type II that could be associated with chronic seizures and base further investigations on cellular protective approaches. Finally, we looked for biomarkers that could contribute to the neuropathological identification of the cell types that characterize FCD type II. Particularly, the differentiation of balloon cells from reactive astrocytes may be difficult considering both morphological and immunohistochemical features (10, 12, 16). Since the presence of balloon cells is necessary to establish the histopathological diagnosis of a type IIb lesion, it is relevant to investigate potential biological markers to support their identification.

## 2. Materials and methods

### 2.1. Surgical samples and groups

This is a retrospective study for which the samples were obtained in biorepositories from both Institutions (University of Campinas, Brazil, and University Hospital Erlangen, Germany). We obtained these samples from patients without the restriction of age or gender but with previous confirmation that the surgical procedure involved the brain area with clinically and MRI determined FCD. We evaluated only frontal lobes for transcriptome analyses, as this is the most common location for FCD ILAE type II (16). For immunohistochemical validation of transcriptome data, we used mainly frontal specimens including some previously submitted to RNA-Seq protocols with remaining formalin-fixed paraffin-embedded (FFPE) tissue. Since our aim was to validate the findings of each lesion type (IIa and IIb) irrespective of the brain area, we included available extrafrontal IIb samples with unquestionable histopathological diagnosis but stored as FFPE tissue only. Moreover, we obtained control samples from frontal lobes of adults submitted to autopsy (between 6 and 12 h *post mortem*) without neurological disease, history of seizures or macro/microscopic brain abnormalities. Details on clinical information and allocation of surgical and control specimens for molecular and histopathological analyses are presented in Tables 1, 2.

**Table 1:** Clinical data of the individuals with diagnosis of FCD ILAE Type II, whose surgical samples were submitted for transcriptome analysis and/or immunohistochemical validation.

**Group**	**#**	**Gd**	**Age at onset (year range)**	**Age at surgery (year range)**	**Duration of crises (year range)**	**Lobe/side**	**Transcriptome**			**IHC**	
							**G**	**W**	**RIN**	**G**	**W**
FCD IIa	1	F	6–10	11–15	0–5	Fr—R			5.6		
	2	F	0–5	11–15	11–15	Fr—R			N/A		
	3	F	6–10	31–35	21–25	Fr—R			6.3		
	4	M	0–5	16–20	11–15	Fr—R			5.5		
	5	M	0–5	6–10	0–5	Fr—L			N/A		
	6	F	0–5	46–50	46–50	Fr—L			5.6		
	7	M	6–10	21–15	16–20	Fr—L			5.5		
	8	F	0–5	16–20	16–20	Fr—L			5.3		
	9	F	0–5	6–10	6–10	Fr—L			6.0		
	10	F	0–5	11–15	11–15	Fr—R			N/A		
	11	F	6–10	31–35	26–30	Fr—R			N/A		
FCD IIb	12	M	0–5	21–25	21–25	Fr—L			N/A		
	13	F	11–15	21–25	6–10	Fr—L			6.3		
	14	F	0–5	0–5	0–5	Fr—L			7.5		
	15	F	0–5	11–15	11–15	Fr—R			5.6		
	16	F	0–5	6–10	0–5	Fr—R			N/A		
	17	F	11–15	41–45	26–30	Fr—L			7.4		
	18	F	11–15	31–35	16–20	Fr—L			4.6		
	19	F	11–15	41–45	26–30	Fr—R			5.1		
	20	M	0–5	16–20	17–20	Fr—R			N/A		
	21	M	6–10	41–45	6–10	Pr—R			N/A		
	22	F	0–5	31–35; 41–45	26–30; 36–40	T—R			N/A		
	23	M	6–10	36–40	31–35	Fr - R			N/A		
	24	F	0–5	26–30	21–25	Pr - R			N/A		
	25	F	16–20	30–35	11–15	O - L			N/A		

All samples submitted for transcriptome analysis were obtained from the frontal lobe, which was defined as the epileptogenic zone after clinical and neuroimaging investigation. Each rectangle colored in gray in the “Transcriptome” and “IHC” columns indicates the technique performed with the corresponding sample from the same individual. For transcriptome analyses, nine (n = 9; individuals #1–9) and 8 (n = 8; individuals #12–19) specimens were obtained for FCD IIa and IIb, respectively. Regarding gray matter investigation, seven (n = 7) specimens were used for FCD IIa and six (n = 6) for IIb. Assessment of the white matter was performed in seven (n = 7) samples of each FCD II type. Please note that, dissected samples from individuals #2, #5, #7, #12, #16, and #18 could only be used for either gray other white matter analysis due to available amount or technical issues (alignment ratio of the generated sequences with the reference genome was less than 80% and 50% for gray and white matter, respectively). Materials from individuals #10, #11 and #20–25 were used for immunostainings only. As regards individual #22, only the surgical sample corresponding to the first surgical procedure (year range 31–35) was used in the current study.

F, female; FCD, focal cortical dysplasia; FCD IIa, FCD International League Against Epilepsy (ILAE) Type IIa; FCD IIb, FCD International League Against Epilepsy (ILAE) Type IIb; Fr, frontal; G, gray matter (cortical layer); IHC, immunohistochemistry; Gd, gender; L, left; M, male; N/A, Not applicable (sample not submitted to transcriptome analysis); O, occipital; Pr, parietal; R, right; Surgery, age at surgery (years); RIN, RNA integrity number of the gray matter of samples submitted to RNA-Seq (please refer to item 2.3 for further details on the white matter specimens); T, temporal; W, white matter; #, number of the individual in the present study.

**Table 2:** Clinical data of the controls, whose samples were submitted for transcriptome analysis and/or immunohistochemical validation.

**Group**	**#**	**Country**	**Gd**	**Age range at death (years)**	**Lobe**	**Transcriptome**			**IHC**	
						**G**	**W**	**RIN**	**G**	**W**
Control	26	Brazil	M	50–55	Fr			5.9		
	27	Brazil	F	36–40	Fr			4.6		
	28	Brazil	F	41–45	Fr			5.0		
	29	Brazil	M	66–70	Fr			5.3		
	30	Brazil	F	31–35	Fr			N/A		
	31	Brazil	M	56–60	Fr			N/A		
	32	Brazil	M	61–65	Fr			N/A		
	33	Brazil	F	31–35	Fr			N/A		

All samples were obtained from the frontal lobe. Gross and microscopic evaluations showed no alteration in both gray and white matters. Each rectangle colored in gray in the “Transcriptome” and “IHC” columns indicates the technique performed with the corresponding sample from the same individual. Samples from individuals #26–29 were used for transcriptome (n = 4) investigations of the gray and white matters. Moreover, samples from individuals #27–33 were submitted to immunostaining. Thus, seven (n = 7) specimens were used for gray and white matter analyses.

F, female; Fr, frontal; G, gray matter (cortical layer); IHC, immunohistochemistry; Gd, gender; M, male; N/A, Not applicable (sample not submitted to transcriptome analysis); RIN, RNA integrity number of the gray matter of samples submitted to RNA-Seq (please refer to item 2.3 for further details on the white matter specimens); W, white matter; #, number of the individual in the present study.

### 2.2. Brain sampling and neuropathological diagnoses

Fresh brain samples were collected from surgical and autopsy specimens and (i) FFPE or (ii) snap frozen in liquid nitrogen and stored at −80°C.

FFPE samples were submitted to diagnostic routine, that is, evaluation of the cortical cytoarchitecture and cellularity and myelination of the white matter in serial 4 μm-sections stained with hematoxylin and eosin (H&E) and submitted to immunohistochemical reactions. For the latter protocol, the sections were exposed to antibodies against NeuN (neuronal marker; 1:1,000, clone A60, Merck Millipore, cat#MAB377, Temecula, CA, USA), MAP2 (neuronal marker; 1:1,000, clone M13, Thermo Fisher, cat#13-1500, Waltham, MA, USA), SMI 32 (neuronal marker; 1:2,500, clone SMI 32, Biolegend, cat#SMI-32R, San Diego, CA, USA), GFAP (astrocytic marker, 1:100, clone 6F2, Dako/Agilent, cat#M0761, Santa Clara, CA, USA), vimentin (1:100, clone V9, e-Bioscience/Thermo Fisher, cat#14-9897-82, Waltham, MA, USA), CD34 (1:50, clone QBEnd-10, Dako, cat#M7165, Glostrup, Denmark) and CNPase (myeloarchitecture marker; 1:500, clone 11-5 B, Millipore, cat#MAB326, Darmstadt, Germany), for 18 h at 4°C. Then, a detection solution containing the secondary antibody and peroxidase (AdvanceTMHRP^®^, Dako, cat#K4068, Glostrup, Denmark; or EnvisionTM Flex+, Dako, cat#K8002, Glostrup, Denmark) was added for 30 min at 37°C. 3,3-diaminobenzidine (DAB) was used as chromogenic substrate and counterstaining was performed with hematoxylin. Negative controls (without primary antibody) were run concurrently with all immunohistochemical reactions.

Samples exhibiting cortical dyslamination, hypertrophic and dysmorphic neurons [disoriented neurons with anomalous cytoplasmic distribution of Nissl substance and accumulation of non-phosphorylated neurofilament (SMI 32-positive)] without or with balloon cells (large cells with opaque eosinophilic cytoplasm, vesicular nucleus and immunopositivity for vimentin) were classified as FCD ILAE type IIa or IIb, respectively (16). Samples from autopsies (control group) were submitted to the same protocol to exclude microscopic alterations. All histological analyses were performed at regions of gyri perpendicularly cut to the pial surface, as recommended by the FCD ILAE Classification (16).

### 2.3. Isolation of cortex and white matter and transcriptome analysis

Frozen samples corresponding to previously evaluated FFPE sections were serially cut (40 μm), mounted in PEN membrane covered slides (Life Technologies), immediately stained with Cresyl Violet and dehydrated with an ethanol series. The gray (cortical layer) and white matters were mechanically dissected from 3 to 4 sections per sample with the aid of a scalpel, collected in RNAse-free individual tubes and stored at −80°C.

Total RNA from each dissected sample was extracted and purified with Trizol (Thermo Fisher Scientific, cat # 15596018, Waltham, MA, USA). Particularly, as the gray matter constituted the major part of each specimen, it was possible to determine the RNA integrity number (RIN) only for the gray matter samples. As RIN values ranged from 4.6 to 7.5, we performed the ribosomal depletion method for RNA-Seq library construction in order to minimize bias related to RNA degradation. Moreover, control tisse samples were obtained from the same brain region considering the surgical specimens (frontal lobe). Then, cDNA libraries were prepared from extracted RNA (200 ng) by using the TruSeq Stranded total RNA kit (Illumina, San Diego, CA, USA) and submitted to next-generation sequencing in a HiSeq 2500 platform (Illumina, San Diego, CA, USA) in High-Output mode, producing 100-bp paired-end sequences. The amount (total number and number of sequences produced per sample) and quality (% of bases over Q30) of the generated sequences were also assessed. Sequence alignment was performed with STAR (https://github.com/alexdobin/STAR) to *Homo sapiens* GRCh37/hg19 assembly.

The DESeq2 (http://www.bioconductor.org/packages/release/bioc/html/DESeq2.html) package was used for transcriptome analyses. A list of differentially expressed genes with a statistical significance set at a *p* < 0.05 (after correction for multiple tests, i.e., adjusted *p*-value) was generated. Such list was used for gene ontology analysis (calculation of enrichment of pathways for the set of differentially expressed genes) by using Panther Classification System 17.0 (17, 18). The results regarding the expression of genes of interest were presented as fold change, a simplified designation that refers to the log_2_Fold change calculation performed by the DESeq2 package when comparing the different groups with each other.

### 2.4. Immunohistochemical detection of proteins encoded by differentially expressed genes

For validation of the transcriptome results, 4 μm-thick sections from FFPE specimens were submitted to immunohistochemical reactions. The sections were incubated with primary antibodies against 3-Hydroxy-3-Methylglutaryl-CoA Synthase 1 (HMGCS1), 3-Hydroxy-3-Methylglutaryl-CoA Reductase (HMGCR), Squalene Epoxidase (SQLE) and glycoprotein non-metastatic melanoma protein B (GPNMB) (please refer to item 3.3) for 18 h at 4°C. Specifications of each antibody are as follows: HMGCS1 (1:500, polyclonal, Abcam, cat# ab155787, Cambridge, United Kingdom), HMGCR (1:300, clone CL0260, Abcam, cat# ab242315, Cambridge, United Kingdom), SQLE (1:300, polyclonal, Sigma-Aldrich/Merck, cat#HPA020762, Darmstadt, Germany) and GPNMB (1:100, clone SP299, Abcam, cat# ab227695, Cambridge, United Kingdom). Afterwards, the detection system containing secondary antibody and peroxidase (AdvanceTMHRP^®^, Dako, cat # K4068, Glostrup, Denmark) was added for 30 min at 37°C. DAB was used as chromogenic substrate and hematoxylin as counterstain. Negative controls (without primary antibody) were run concurrently with all reactions.

Following the immunohistochemical reactions, all slides were scanned by using an Aperio Scanscope CS2 (#23CS100) device. Ten representative pictures of each slide, at a 200× magnification, were chosen for the quantification of each marker, which was performed in the ImageJ^®^ software environment. The IHC Profiler plugin (19) was applied to each image in the deconvolution step, that is, separation of hematoxylin and DAB channels. The corresponding DAB channel picture was used for quantification *via* the “threshold” tool. A threshold value was determined so that the histological findings in each picture were preserved in the most accurate way considering the original photo. The final image consisted of a black-and-white picture from which the percentage of positive pixels was obtained.

The Kruskal-Wallis test and the *post-hoc* Dunn tests were performed with the GraphPad Prism software (version 8.0) for statistical analyses. Statistical significance was set at a *p* < 0.05.

## 3. Results

### 3.1. Clinical and neuropathological data

**Figure 1:**
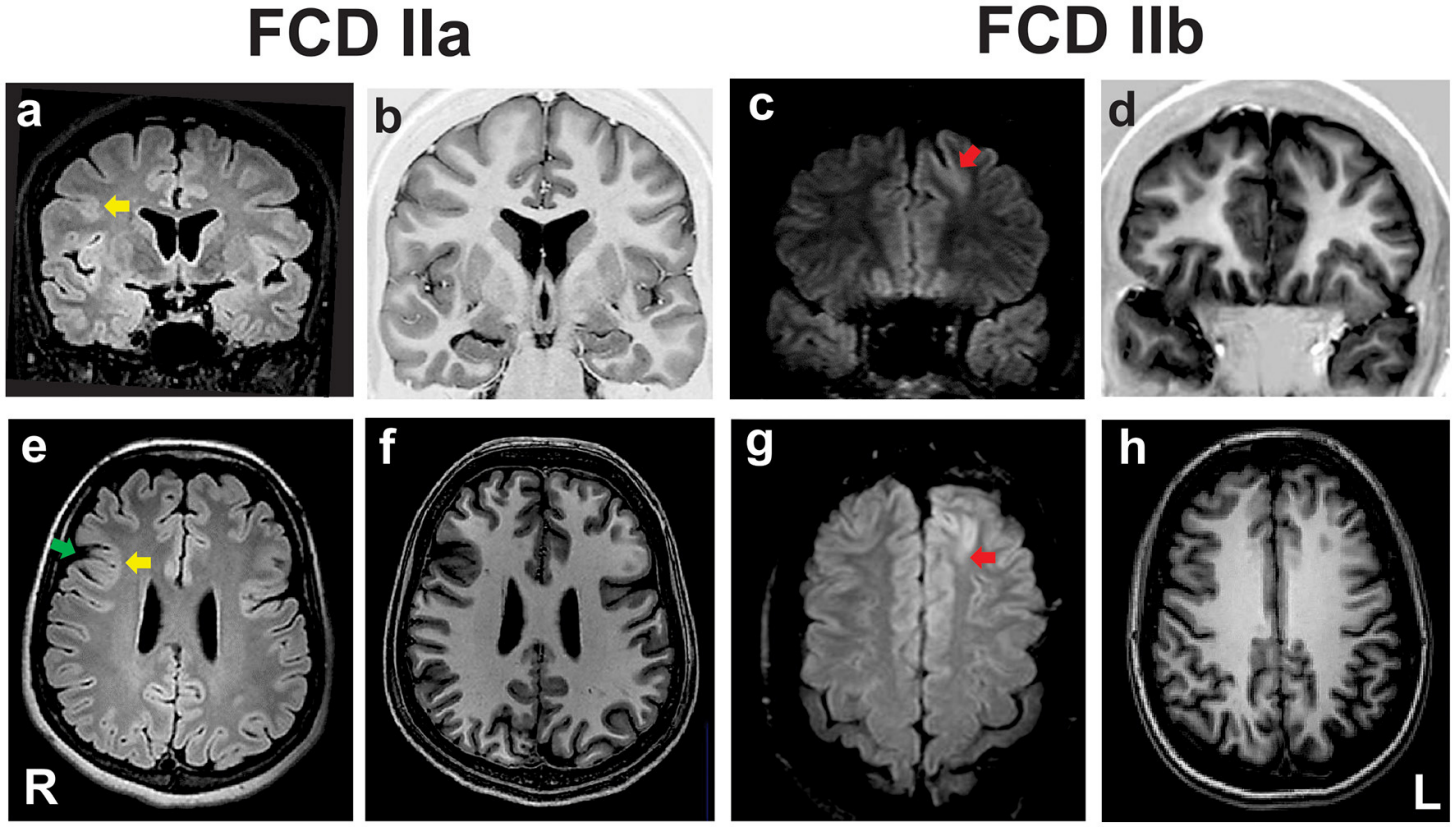
Preoperative coronal fluid-attenuated inversion recovery [FLAIR; **(a, c)**] and T1 inversion recovery **(b, d)**, and axial FLAIR **(e, g)** and T1-weighted images **(f, h)** of two patients with pharmacoresistant frontal lobe epilepsy whose surgical samples showed focal cortical dysplasia (FCD) ILAE type IIa **(a, b, e, f)** and FCD type IIb **(c, d, g, h)**. In the patient with FCD IIa there is a discrete hyperintense signal of gray matter in T2/FLAIR [yellow arrows in **(a, e)**], mild blurring of the gray-white matter junction in T1 scans, and a cortical dimple indicated by the green arrow in **(e)**. The patient with FCD type IIb has a deep sulcus with a mildly thickened cortex and hyperintense FLAIR signal in the left frontal lobe [red arrows in **(c, g)**]. ILAE, International League Against Epilepsy; L, left side; R, right side.

Patients whose samples were histopathologically assigned as FCD ILAE type IIa or FCD ILAE type IIb were submitted to presurgical evaluation consisting of routine EEGs, video-EEG monitoring, neuropsychological assessment, magnetic resonance imaging (MRI), and Fluorodeoxyglucose-Positron Emission Tomography (FDG-PET) and ictal Single-Photon Emission Computed Tomography (SPECT) studies when indicated. Information on the number of individuals per group and clinical data regarding the origin (country), gender, age at epilepsy onset, age at surgery and epilepsy duration is shown in Table 1. All patients presented MRI features of FCD type II that included focal cortical thickening, a varying degree of increased cortical and subcortical signal intensity in T2-weighted and Fluid Attenuated Inversion Recovery (FLAIR) sequences, blurring of the gray-white matter junction, focal abnormal cortical gyration, and cerebrospinal fluid cleft-cortical dimple (Figure 1). The hyperintense T2-FLAIR signal in the subcortical white matter with a wedge shape extending to the ipsilateral ventricle ependymal surface (transmantle sign) was present in some but not all patients with FCD type IIb.

**Figure 2:**
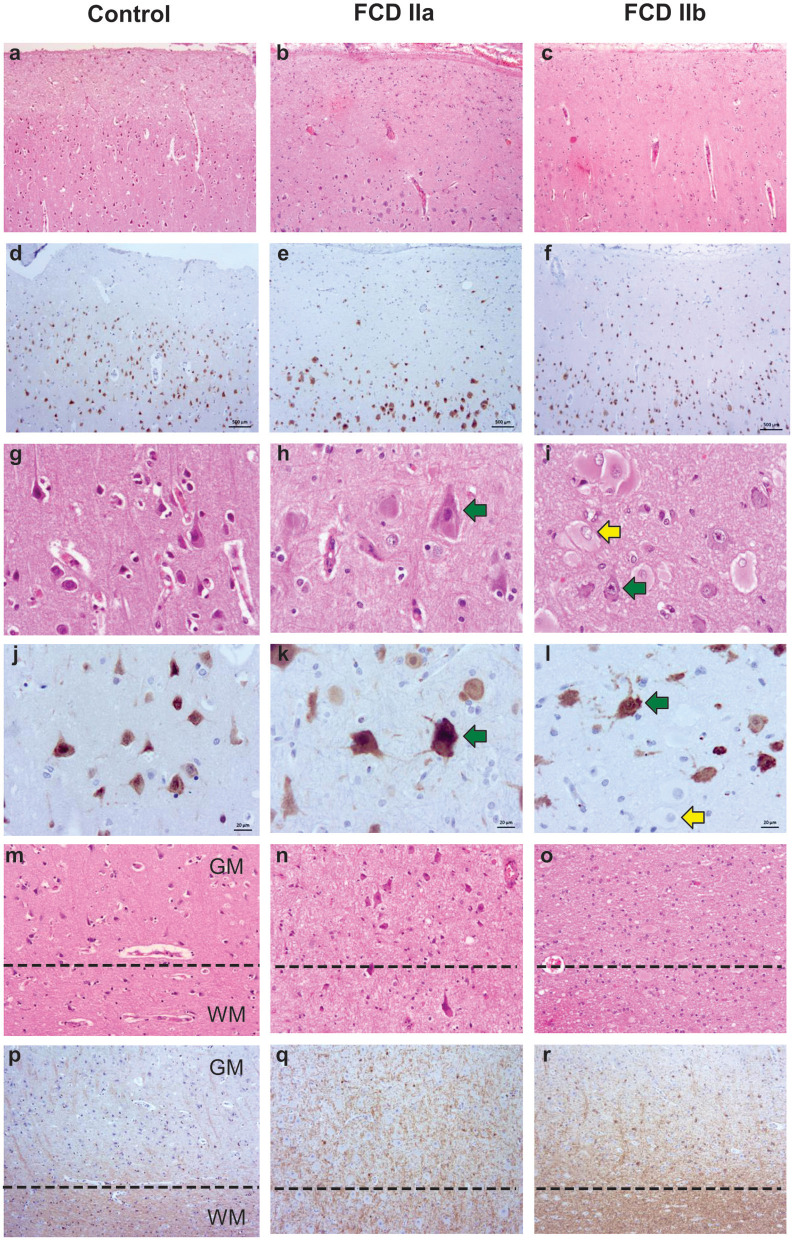
Representative histopathological and immunostaining features of autopsy (control), focal cortical dysplasia (FCD) ILAE type IIa (FCD IIa) and FCD ILAE type IIb (FCD IIb) samples. Hematoxylin and eosin staining **(a–c, g–i, m–o)**. Immunohistochemistry for NeuN **(d–f, j–l)** or CNPase **(p–r)**. Cortical cytoarchitectural features, that is, layering and neuronal size and orientation, are preserved in controls **(a, d, g, j, m)** specimens. However, dyslamination **(b, c, e, f, h, i, k, l, n, o)** and dysmorphic neurons [green arrows in **(h, i, k, l)**] without and with balloon cells [yellow arrows in **(i, l)**] are observed in FCD IIa and FCD IIb, respectively. Gray matter (GM) and white matter (WM) boundary [dotted line in **(m–o)** and **(p–r)**] is preserved in control samples **(m, p)** and blurred in FCD IIa **(n, q)** and FCD IIb **(o, r)**. Scale bars: 500 μm **(a–f)**, 20 μm **(g–l)**, and 50 μm **(m–r)**.

The neuropathological evaluation revealed samples with either cortical dyslamination and dysmorphic neurons (FCD IIa diagnosis) or cortical dyslamination, dysmorphic neurons and balloon cells (FCD IIb diagnosis). Gliosis and gray-white matter blurring in both FCD types were shown by increased GFAP positivity and irregularly distributed CNPase immunostaining, respectively. Heterotopic and dysmorphic neurons, as well as balloon cells, were also observed in the white matter of FCD II samples (Figure 2). Moreover, immature CD34-positive cells were detected neither in the gray nor in the white matter.

Control specimens were obtained from autopsied individuals without a history of neurological diseases (clinical information is presented in Table 2). Histological evaluation showed neither cortical dyslamination nor cytological abnormalities in the gray and white matter (Figure 2).

### 3.2. Transcriptome analysis

Seventeen FCD specimens from the UNICAMP (Brazil) and FAU (Germany) biorepositories were assessed in the transcriptome study (Table 1). Brazilian and German samples classified as FCD IIa were considered as one group for gene expression analyses, the same for FCD IIb specimens. Other four samples from the UNICAMP biorepository were used as controls (Table 2).

**Figure 3:**
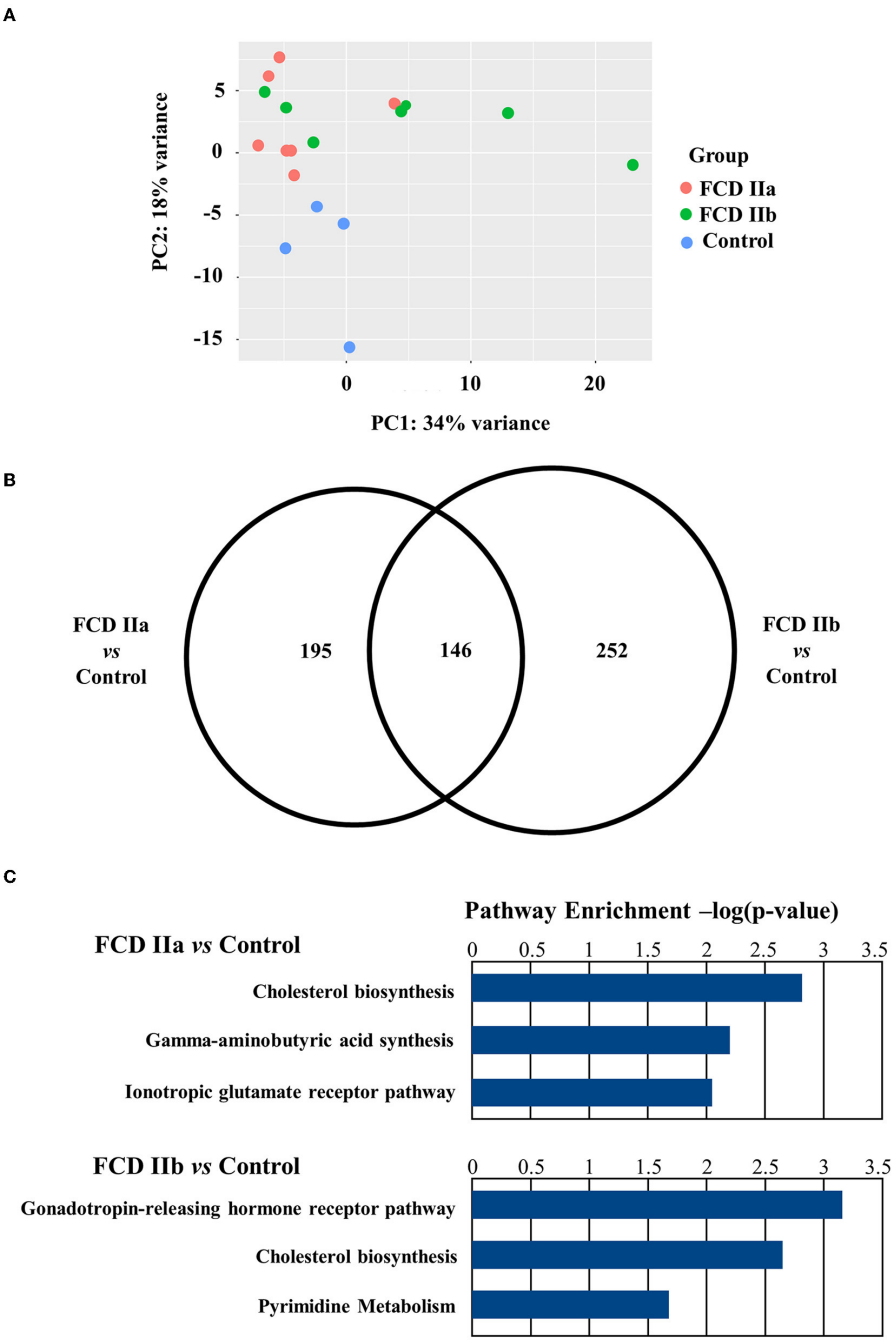
Gene expression analyses of the gray matter (cortical layer) of autopsy (control), focal cortical dysplasia (FCD) ILAE type IIa (FCD IIa) and FCD ILAE type IIb (FCD IIb) samples. **(A)** Graphical presentation of gene expression data after analyses performed with the PCA dimensionality reduction method. Brazilian and German FCD specimens with the same histopathological classification are shown as one group (FCD IIa or FCD IIb). Note that the majority of FCD IIa samples tended to group together, as observed for FCD IIb cases. Each circle corresponds to an individual sample. **(B)** Venn diagram showing the number of differentially expressed genes in each group compared to controls. FCD IIa and FCD IIb groups showed 342 and 399 differentially expressed genes, respectively. A total of 146 genes were common to FCD IIa and IIb groups. The highest number of exclusively differentially regulated genes was verified in FCD IIb (252). **(C)** Graphs depicting the three main enriched pathways containing the highest numbers of differentially expressed genes represented in the Venn diagram for FCD IIa and FCD IIb (vs. control group). Values presented in the X-axis correspond to Enrichr –log(*p*-value) by using Panther Pathways (version 17.0). Pathways were considered statistically significant when associated with values higher than –log(0.05) = 1.30. Additional data on enriched pathways and details on gene expression are reported in the Supplementary material.

Regarding the gray matter (cortical layer), a total of 342 and 399 differentially expressed genes were identified after comparing FCD IIa and FCD IIb to controls, respectively. In the comparison between FCD IIa and FCD IIb samples, we found 12 genes differentially expressed. Moreover, a principal component analysis (PCA) was computed on the rlog transformation of the count matrix generated by the STAR aligner, demonstrating that FCD IIa and FCD IIb samples aggregated in distinct clusters (Figure 3; Table 3).

**Table 3:** Number of differentially expressed genes of the gray matter (cortical layer) comparing the autopsy (control), focal cortical dysplasia (FCD) ILAE type IIa (FCD IIa) and FCD ILAE type IIb (FCD IIb) groups.

**Groups**		**Number of differentially expressed genes (adjusted p-value < 0.05)**
Control vs. (*n* = 4)	FCD IIa (*n* = 7)	342
	FCD IIb (*n* = 6)	399
FCD IIa vs. (*n* = 7)	FCD IIb (*n* = 6)	12

An overrepresentation pathway analysis was performed for the differentially expressed genes in FCD IIa or FCD IIb vs. the control group by using the Panther Classification System 17.0. The main pathways found considering the Panther 17.0 Pathways dataset were Cholesterol biosynthesis, Gamma-aminobutyric acid synthesis, and Ionotropic glutamate receptor pathway for FCD IIa. In FCD IIb, the main enriched pathways were Gonadotropin-releasing hormone receptor pathway, Cholesterol biosynthesis, and Pyrimidine Metabolism (Figure 3; enriched pathways and the corresponding differentially expressed genes are presented as Supplementary material).

**Figure 4:**
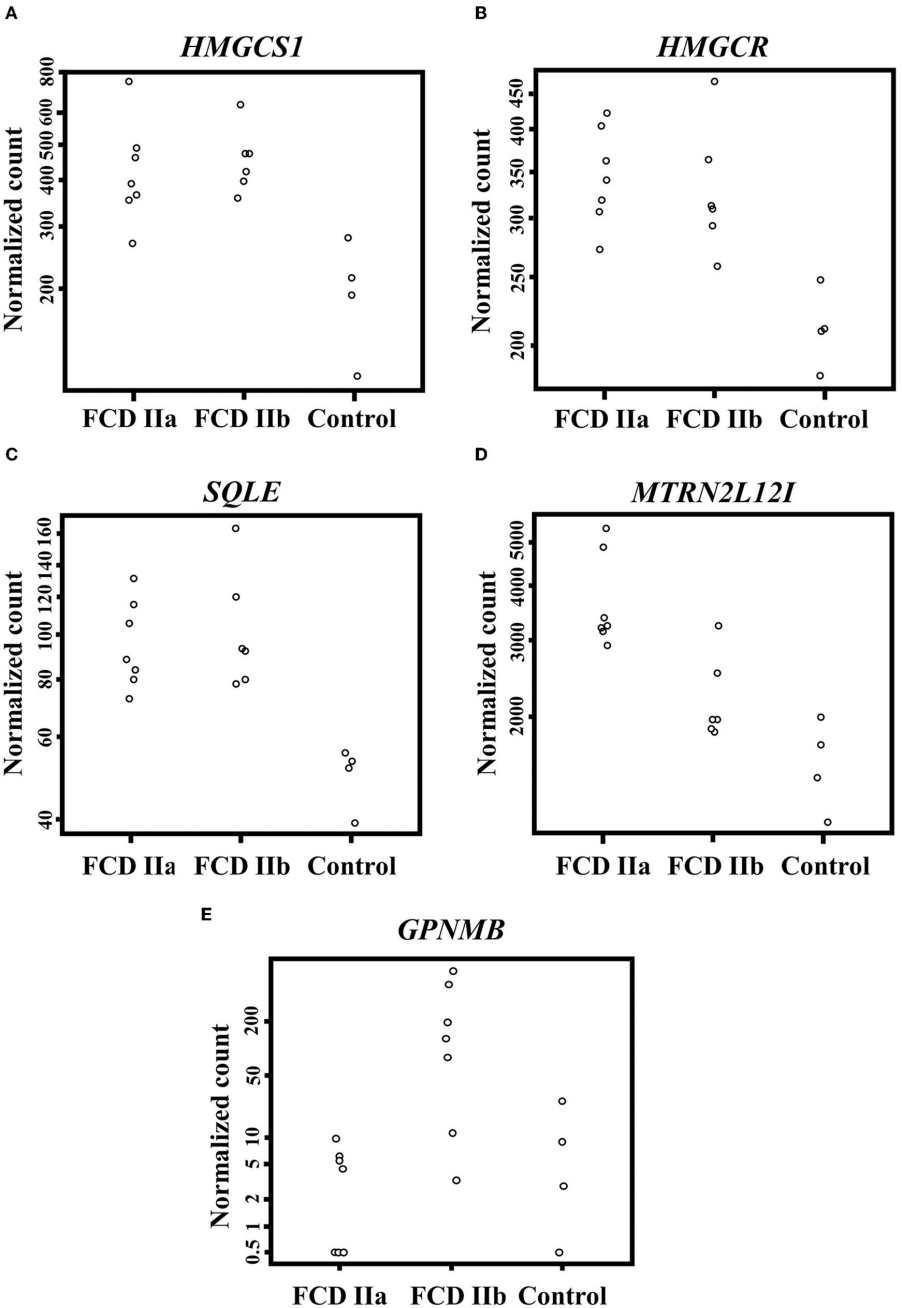
Differentially expressed genes when comparing the autopsy (control), focal cortical dysplasia (FCD) ILAE type IIa (FCD IIa) and FCD ILAE type IIb (FCD IIb) groups. **(A–D)** Expression of *HMGCS1, HMGCR, SQLE*, and *MTRNR2L12*, respectively, in the gray matter. **(E)** Expression of *GPNMB* in the white matter. Please refer to the text for details on fold change for each gene and the corresponding statistical significance (adjusted *p*-value).

When taking into account genes less frequently or not previously investigated in human FCDs, we found upregulation of three cholesterol biosynthesis genes in FCDs, namely *HMGCS1* (3-Hydroxy-3-Methylglutaryl-CoA Synthase 1; FCD IIa vs. Control: Fold change = 0.92, adjusted *p*-value = 0.001; FCD IIb vs. Control: Fold change = 0.98, adjusted *p*-value = 0.001); *HMGCR* (3-Hydroxy-3-Methylglutaryl-CoA Reductase; FCD IIa vs. Control: Fold change = 0.63, adjusted *p*-value = 0.005; FCD IIb vs. Control: Fold change = 0.59, adjusted *p*-value = 0.01); and *SQLE* (Squalene Epoxidase; FCD IIa vs. Control: Fold change = 0.76, adjusted *p*-value = 0.01; FCD IIb vs. Control: Fold change = 0.89, adjusted *p*-value = 0.002) (Figure 4).

Moreover, a subsequent analysis focusing on the differentially expressed genes between FCD IIa and FCD IIb showed that *MTRNR2L12* was the only upregulated transcript in FCD IIa (FCD IIa vs. Control: Fold change = 1.09, adjusted *p*-value = 0.0000003; FCD IIa vs. FCD IIb: Fold change = 0.67; adjusted *p*-value = 0.05) (Figure 4).

**Figure 5:**
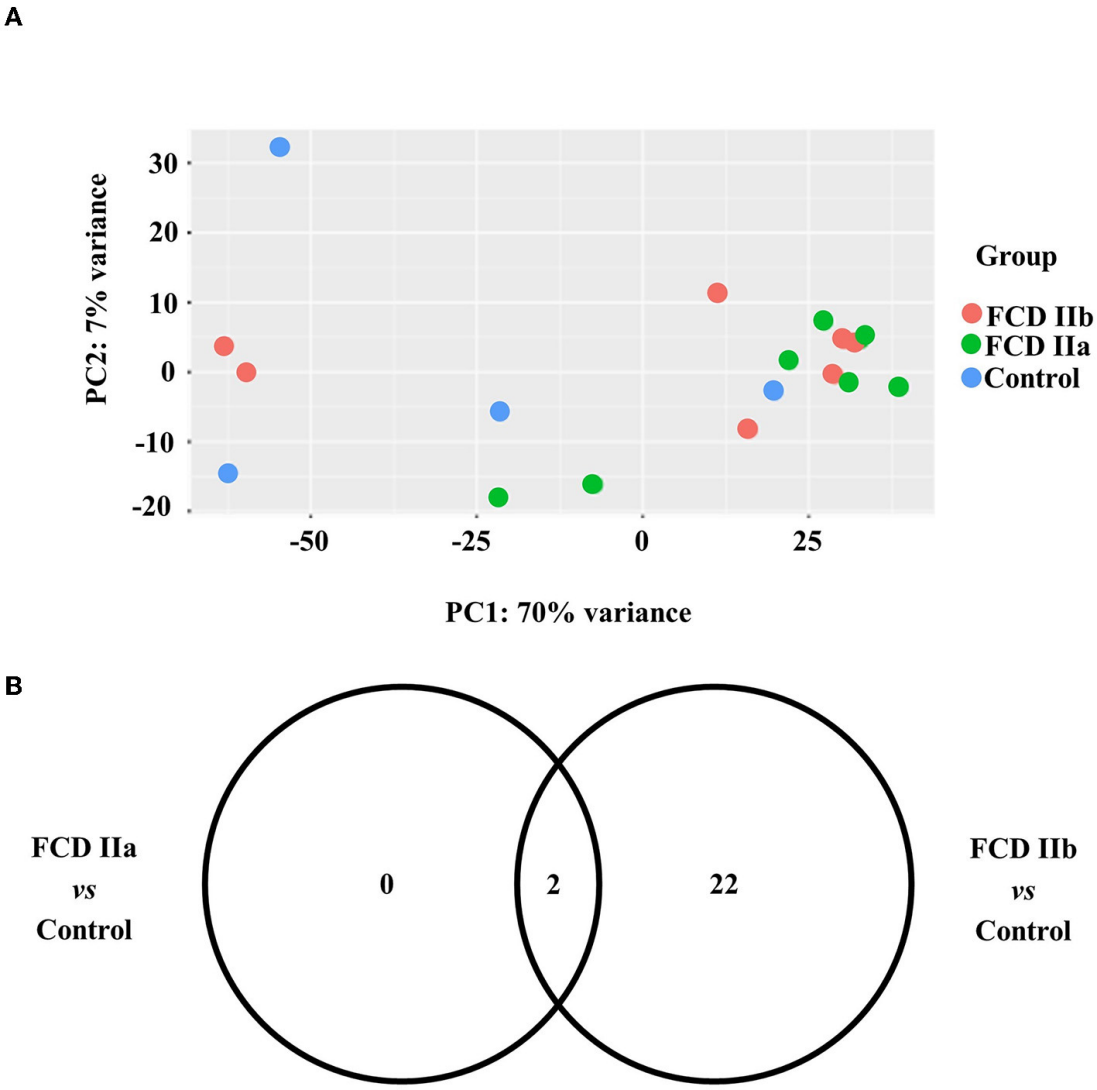
Gene expression analyses of the white matter of autopsy (control), focal cortical dysplasia (FCD) ILAE type IIa (FCD IIa) and FCD ILAE type IIb (FCD IIb) groups. **(A)** Graphical presentation of gene expression data after analyses performed with the PCA dimensionality reduction method. Brazilian and German FCD specimens with the same histopathological classification are shown as one group (FCD IIa or FCD IIb). Samples with the same neuropathological diagnosis did not cluster together. **(B)** Venn diagram showing the number of differentially expressed genes in each group compared to controls. FCD IIa and FCD IIb groups showed 2 and 24 differentially expressed genes, respectively. Two genes were common to FCD IIa and IIb groups. The highest number of exclusively differentially regulated genes was verified in FCD IIb (20). Additional data on gene expression are reported in the Supplementary material.

As regards the white matter, the comparison of FCD IIa and FCD IIb with the control group showed a total of 2 and 24 differentially expressed genes, respectively. When comparing FCD IIa and FCD IIb groups, we found four genes differentially expressed (Figure 5; Table 4; Supplementary material). No clustering of samples was identified by using the PCA method and the corresponding RNA-Seq data. Similarly, the Panther 2022 found no enriched pathways considering the three groups in the pathways dataset. Also, we performed analyses focusing on differentially expressed genes not previously reported in human FCDs. Particularly, *GPNMB* (glycoprotein non-metastatic melanoma protein B) was upregulated in FCD IIb vs. Control (Fold change = 4.50, adjusted *p*-value = 0.03) and downregulated in FCD IIa vs. FCD IIb (fold change = −5.68, adjusted *p*-value = 0.0000124) (Figure 4).

**Table 4:** Number of differentially expressed genes of the white matter comparing the autopsy (control), focal cortical dysplasia (FCD) ILAE type IIa (FCD IIa) and FCD ILAE type IIb (FCD IIb) groups.

**Groups**		**Number of differentially expressed genes (adjusted *p*-value < 0.05)**
Control vs. (*n* = 4)	FCD IIa (*n* = 7)	2
	FCD IIb (*n* = 7)	24
FCD IIa vs. (*n* = 7)	FCD IIb (*n* = 7)	4

### 3.3. Immunohistochemical validation of the transcriptome results

In this step, we used a total of 20 samples from Brazilian individuals (4 FCD IIa, 9 FCD IIb, and 7 controls; Tables 1, 2). Considering the differential expressions of *HMGCS1, HMGCR, SQLE*, and *GPNMB* in FCD groups, we further evaluated immunohistochemically the expression of the encoded proteins by using available commercial antibodies.

#### 3.3.1. HMGCS1, HMGCR, and SQLE

**Figure 6:**
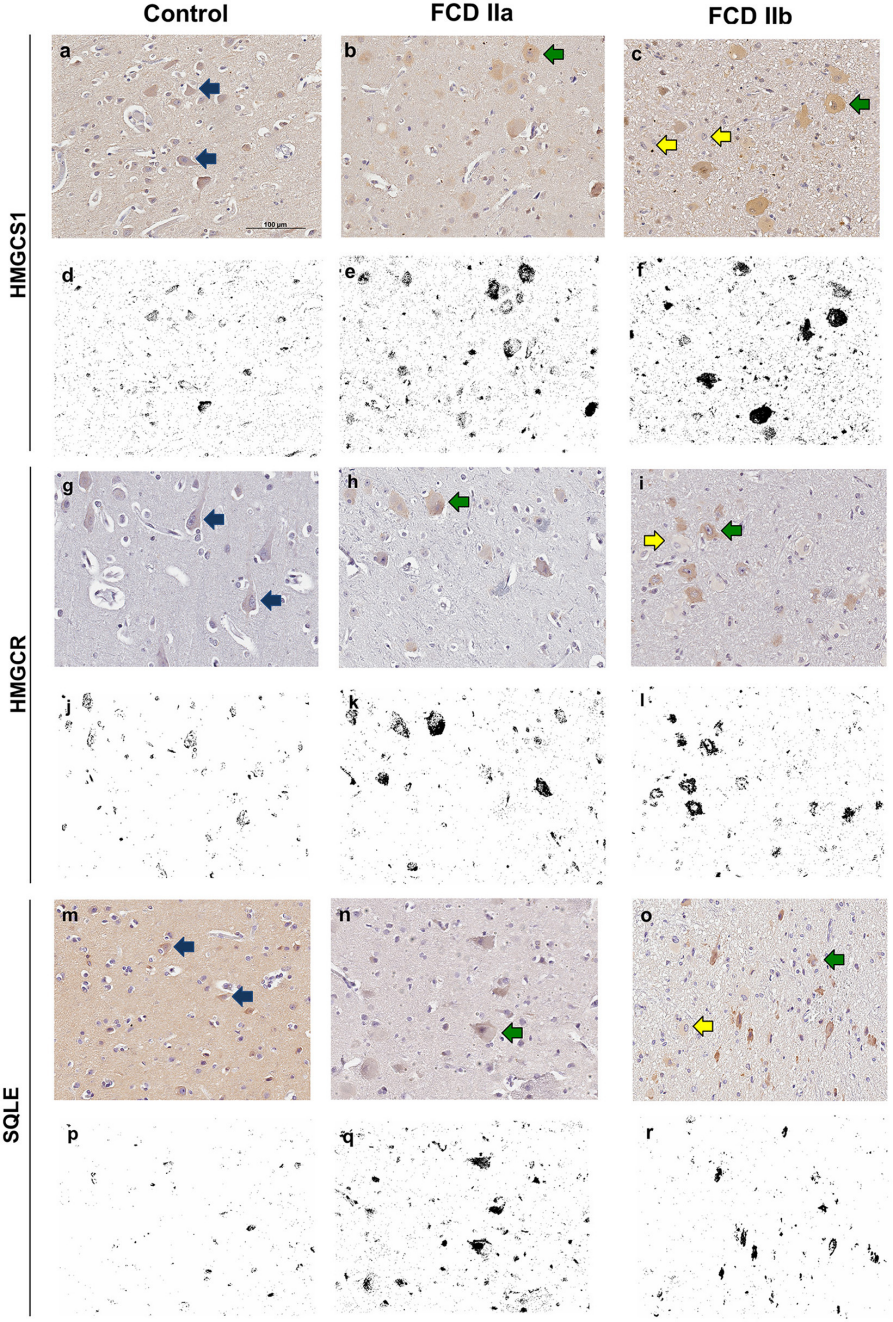
Immunohistochemical reactions for 3-Hydroxy-3-Methylglutaryl-CoA Synthase 1 (HMGCS1), 3-Hydroxy-3-Methylglutaryl-CoA Reductase (HMGCR) and Squalene Epoxidase (SQLE) in the cortical layer of control **(a, g, m)**, focal cortical dysplasia (FCD) ILAE type IIa (FCD IIa) **(b, h, n)** and FCD ILAE type IIb (FCD IIb) **(c, i, o)** specimens. In FCD IIa and FCD IIb, a cytoplasmic staining was observed in dysmorphic neurons [green arrows in **(b, c, h, i, n, o)**], in contrast with the negativity in balloon cells of FCD IIb lesions [yellow arrows in **(c, i, o)**]. Normal neurons [blue arrows in **(a, g, m)**] showed less noticeable cytoplasmic positivity compared to the dysmorphic neurons. Digital images of the presented immunohistochemical reactions were obtained by using the ImageJ^®^ software **(d–f, j–l, p–r)**. In this setting, **(d–f, j–l, p–r)** correspond to the images shown in **(a–c, g–i, m–o)**, respectively, after deconvolution and threshold steps. Note that the original immunohistochemical findings (stained structures and tissue distribution) were preserved. Scale bar: 100 μm **(a–r)**.

Immunostaining for the cholesterol biosynthesis enzymes HMGCS1, HMGCR and SQLE showed a diffuse cytoplasmic pattern, mainly observed in normal and dysmorphic neurons. Particularly, in FCD groups, immunopositive abnormal neurons were more frequently observed than their normal counterparts. Balloon cells were virtually immunonegative (Figure 6).

**Figure 7:**
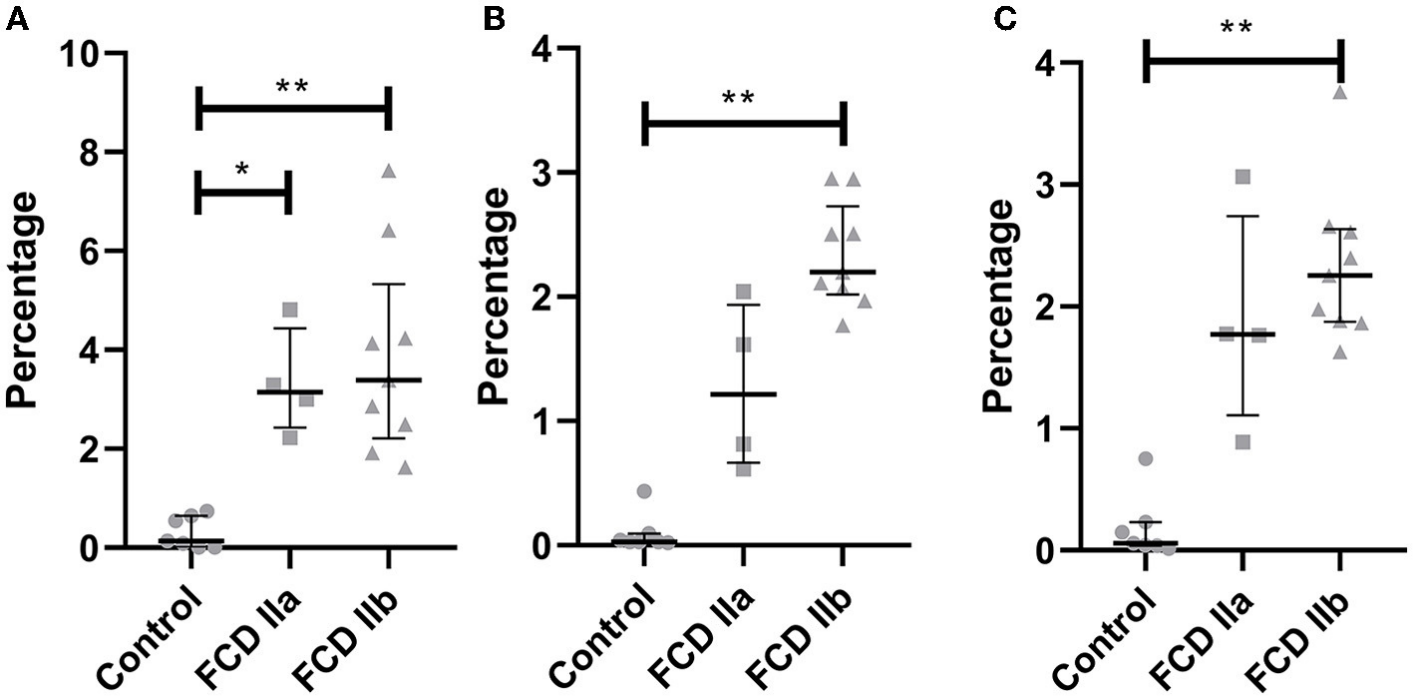
Quantification of the immunohistochemical expressions (percentage of positive pixels per total number of pixels) of HMGCS1 **(A)**, HMGCR **(B)** and SQLE **(C)** in the gray matter of focal cortical dysplasia and controls. The graphs show scatterplots with superimposed median and interquartile range (25–75). *p < 0.05, **p < 0.01 (Kruskall-Wallis test followed by Dunn's *post-hoc* test).

Quantitative evaluation of HMGCS1 immunostaining by using the ImageJ^®^ software yielded the values 0.31 ± 0.32 (mean percentage value ± standard deviation), 3.33 ± 1.08, and 3.86 ± 2.02 for controls, FCD IIa and FCD IIb samples, respectively. Significantly higher expressions were found in FCD IIa and FCD IIb compared to controls (*p* = 0.02 and 0.002, respectively). No difference was found after comparing FCD IIa and FCD IIb groups (*p* = 0.99) (Figures 6, 7).

As regards HMGCR, the values for controls, FCD IIa and FCD IIb were 0.10 ± 0.15, 1.27 ± 0.67, and 2.34 ± 0.42, respectively. A higher expression was observed in FCD IIb when compared to controls (*p* = 0.0002). No differences were found when comparing controls and FCD IIa (*p* = 0.31), as well as FCD IIa and IIb groups (*p* = 0.31) (Figures 6, 7).

Values for SQLE immunostaining were 0.18 ± 0.26, 1.87 ± 0.90, and 2.34 ± 0.64 for controls, FCD IIa and FCD IIb groups, respectively. A higher expression of SQLE was found when comparing FCD IIb with controls (*p* = 0.0008). However, no differences were found between FCD IIa and controls (*p* = 0.09), as well as between FCD IIa and IIb (*p* = 0.99) (Figures 6, 7).

Taken together, immunohistochemical detections of HMGCS1, HMGCR, and SQLE paralleled most of the upregulation of the corresponding transcripts in samples with FCD, as compared to controls.

#### 3.3.2. GPNMB

**Figure 8:**
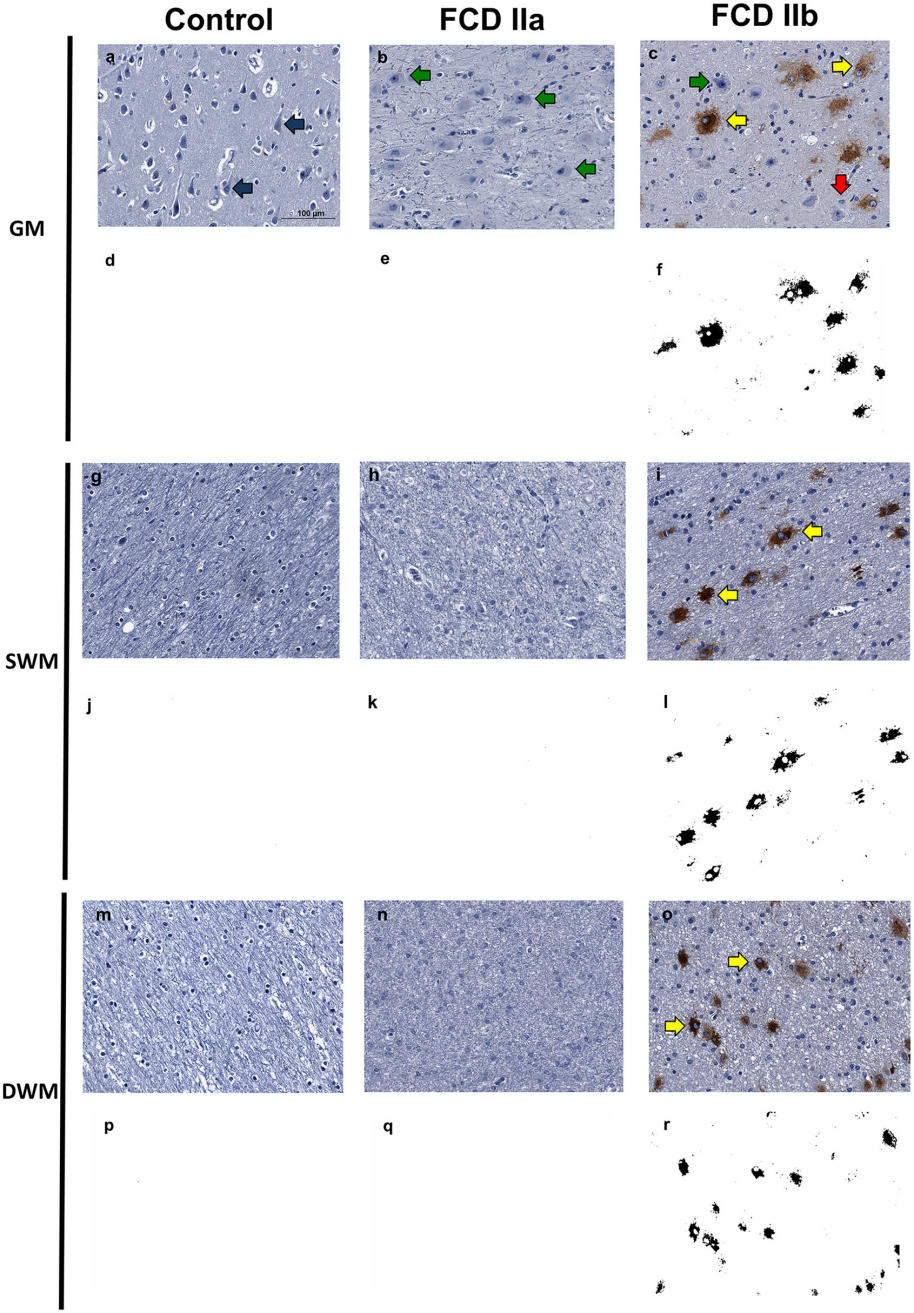
Immunohistochemical reaction for GPNMB in control, focal cortical dysplasia (FCD) ILAE Type IIa (FCD IIa) and FCD ILAE Type IIb (FCD IIb) specimens. The gray matter (GM), superficial white matter (SWM) and deep white matter (DWM) are shown in **(a–f, g–l, m–r)**, respectively. Immunopositivity was noted virtually only in balloon cells. Particularly, the staining in such cells varied from null [red arrows in **(c)**] to intense [yellow arrows in **(c, i, o)**]. Both dysmorphic [green arrows in **(b, c)**] and normal [blue arrows in **(a)**] neurons were negative. Digital images of the presented immunohistochemical reactions were obtained by using the ImageJ^®^ software **(d–f, j–l, p–r)**. In this setting, **(d–f, j–l, p–r)** correspond to the images shown in **(a–c, g–i, m–o)**, respectively, after deconvolution and threshold steps. Note that the original immunohistochemical findings (stained structures and tissue distribution) were preserved. Scale bar: 100 μm **(a–r)**.

Immunopositivity for GPNMB was noted only in balloon cells. Most of them showed diffuse cytoplasmic staining, which varied from light to dense. Some balloon cells, however, were immunonegative. Normal and dysmorphic neurons showed no reactivity. Similarly, other normal cell types (glia, endothelial, and leptomeningeal) were negative for this marker. Since immunohistochemical reactions were performed in sections containing both gray and white matters, it was possible to identify immunopositive balloon cells in both compartments, with a random distribution but predominantly in subpial and superficial white matter regions (Figure 8).

**Figure 9:**
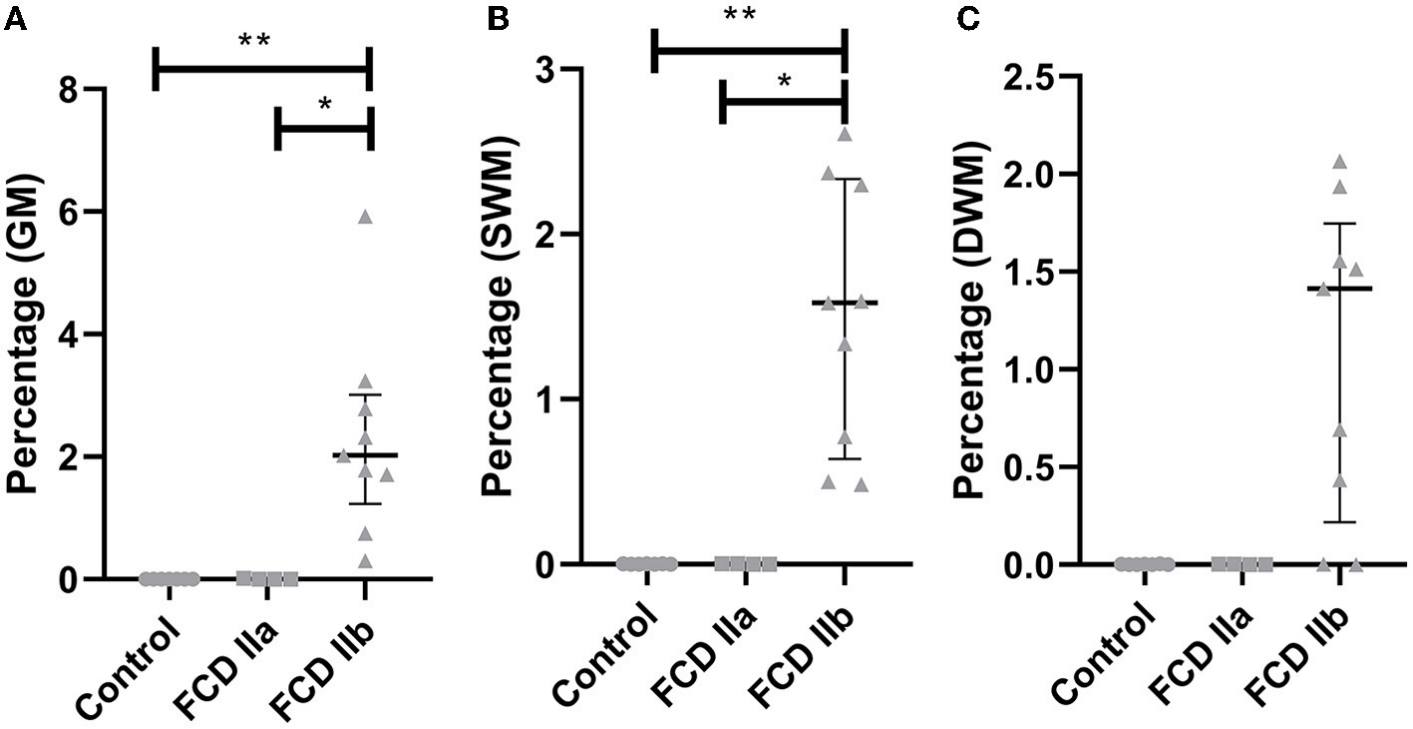
Quantification of the immunohistochemical expression (percentage of positive pixels per total number of pixels) of GPNMB in the gray matter [GM; **(A)**], superficial white matter [SWM; **(B)**] and deep white matter [DWM; **(C)**] of focal cortical dysplasia and controls. The graphs show scatterplots with superimposed median and interquartile range (25–75). *p < 0.05; **p < 0.01 (Kruskall-Wallis test followed by Dunn's *post-hoc* test).

After quantitative assessment of GPNMB positivity by using the ImageJ^®^ software, the following values were verified in the superficial white matter for controls, FCD IIa and FCD IIb groups: 0.002 ± 0.001, 0.003 ± 0.001, and 1.510 ± 0.810, respectively. As regards the deep white matter, the values for controls, FCD IIa and FCD IIb were: 0.002 ± 0.001, 0.002 ± 0.001, and 1.07 ± 0.801, respectively. When considering the superficial white matter, significantly higher expression was found in FCD IIb compared to FCD IIa and controls (*p* = 0.02 and *p* = 0.001, respectively). No difference was found between FCD IIa and controls (*p* = 0.99). At the deep white matter level, no significant differences were found between the groups (FCD IIa vs. controls: *p* = 0.99; FCD IIb vs. controls: *p* = 0.07; FCD IIa vs. FCD IIb: *p* = 0.09) (Figures 8, 9).

The observation of GPNMB-positive balloon cells in the dysplastic cortical layer prompted us to look for the corresponding gene expression in the gray matter transcriptome of each group. Likewise, *GPNMB* was found to be significantly upregulated in FCD IIb vs. controls (Fold change = 1.08, adjusted *p*-value = 0.01) and downregulated in FCD IIa vs. FCD IIb (Fold change = −1.18, adjusted *p*-value = 0.04). *GPNMB* was not present in the list of differentially expressed genes, when FCD IIa and control groups were compared.

Quantitative evaluation of GPNMB immunostaining in the gray matter showed the values 0.002 ± 0.001, 0.005 ± 0.005, and 2.314 ± 1.632 for controls, FCD IIa and FCD IIb samples, respectively. A higher expression was observed in FCD IIb when compared to controls and FCD IIa (*p* = 0.002 and *p* = 0.01, respectively). No difference was found when comparing controls and FCD IIa (*p* = 0.99) (Figures 8, 9).

## 4. Discussion

In this work, we aimed to generate additional insights into the molecular landscape of FCD tissues by assessing the transcriptome profile, enriched cellular pathways, differential gene expression, and protein synthesis in surgical samples from patients with FCD type II. For this purpose, we designed an original protocol to dissect the gray and white matter and evaluate each transcriptome by using a high-throughput sequencing method (RNA-Seq). Such design aimed to avoid biases due to analyses performed with tissue homogenates from both regions of the same sample (21). Overall, our results revealed different transcriptomic signatures in FCD type IIa and IIb lesions. Of note, the RNA-Seq method allowed us to obtain data not considered in previous studies focusing only on histopathological analyses of human FCD samples (13–15). Thus, such genetic data prompted us to further histopathologically and specifically investigate the expressions of cholesterol biosynthesis enzymes and GPNMB, which are respectively potential neuropathological biomarkers of a dysplastic brain cortex chronically exposed to seizures and of balloon cells. Even though we used extrafrontal samples (parietal, temporal or occipital) to immunohistochemically validate transcriptome data regarding type IIb lesions, the histopathological findings were essentially the same in all lobes, thus favoring the association of the current data with FCD type II irrespective of the brain region.

Regarding the gray matter, we found enrichment of a wide range of cellular pathways and the differential expression of 342 and 399 genes, respectively, in IIa and IIb groups compared to controls. Therefore, we focused our discussion on genes not previously described in human FCDs. In this sense, we concentrated on the Cholesterol biosynthesis as this was one of the main enriched pathways of both FCD IIa and IIb. *HMGCS1, HMGCR*, and *SQLE* were selected as such genes showed increased expression in both lesions and we found no previous reports on their expressions in FCD type II. Moreover, we decided to validate our transcriptome results by performing immunohistochemical analyses of the encoded proteins.

Cholesterol is a molecule with a wide range of functions, including cellular signaling and stability, fluidity, and plasma membrane permeability. Cholesterol may be synthesized by either neurons or glial cells in the central nervous system. Briefly, the initial steps involve 3-Hydroxy-3-Methylglutaryl-CoA Synthase 1 (HMGCS1) conjugating acetyl-CoA with acetoacetyl-CoA to synthesize 3-Hydroxy-3-Methylglutaryl-CoA (HMG-CoA), which is the substrate for HMG-CoA Reductase (HMGCR) to produce mevalonate. Subsequently, mevalonate is converted to squalene by Squalene Epoxidase (SQLE). In neurons, cholesterol takes part in synaptogenesis and growth of dendrites and axons (20, 22, 23).

Here, the immunohistochemical analyses of HMGCS1, HMGCR, and SQLE supported most of the upregulation of transcripts detected in FCD types IIa and IIb, compared to controls. Particularly, we observed a higher percentage of immunopositivity for the three enzymes in IIb lesions and HMGCS1 in IIa samples. The fact that the immunohistochemical expression of the downstream enzymes HMGCR and SQLE did not parallel the gene expression in IIa lesions may be due to molecular events associated with transcription and translation processes, including abundances and degradation rates of mRNA and protein, alternative splicing and post-translation changes (24–26). However, such cellular processes did not seem to be discordant regarding the initial steps of cholesterol synthesis in FCD IIa, since HMGCS1 immunoexpression was also higher in this lesion than in controls.

We, therefore, put forward the hypothesis that the enrichment of the cholesterol pathway in the context of chronic seizures due to FCD may be a tissue response to the imbalance in processes such as neurotransmission, synaptic vesicle exocytosis, and maintenance of dendritic spines and axons. Furthermore, since cytotoxic-T cell infiltrates may be observed in tissue from patients with FCD type II (27, 28), cholesterol pathway enrichment may be a response to minimize resident cell damage. Indeed, cholesterol-containing lipoproteins secreted by glia cells exert anti-inflammatory and anti-apoptotic actions on glia and neurons, respectively (20, 22). We consider that our results may contribute with future investigations on the cellular protective role of the proteins HMGCS1, HMGCR and SQLE as well as with experimental approaches to regulate their expressions and/or activities in FCD.

Previous reports addressed the transcriptomic characterization of FCD brain samples. Donkels et al. (13) investigated 19 FCD samples (Ia, IIa, and IIIa) and compared their transcriptome profiles (gray matter only) with non-FCD epileptic controls by using the Affymetrix platform. They found reduced myelin-associated transcripts in FCD samples, probably reflecting long-term damage resulting from epileptogenic stimuli. In 2018, Dixit et al. (14) sequenced three patients with FCD (IIa and IIb), whose transcriptomes were compared with two controls from autopsies (considering transcripts of gray and white matters simultaneously). The authors observed epigenetic regulation in genes involved in neuron cell migration. Moreover, the recent study by Srivastava et al. (15) addressed the transcriptomes of 5 FCDs (IIa and IIb) and 2 controls, also considering gray and white matters concurrently. Interestingly, they found an upregulation of myelin-associated transcripts in FCD cases that had high electric activity, compared with controls from autopsies. In addition, Srivastava et al. (15) found differential expression of lipid-biosynthesis enzymes between FCD and controls, which is similar to our finding concerning cholesterol biosynthesis-enzymes. Despite such similarity, caution is necessary when comparing our data and those from others due to particularities associated with sequencing methods and control selection.

Furthermore, we found that *MTRNR2L12* was the only upregulated transcript in the gray matter of FCD type IIa lesions compared to IIb. To the best of our knowledge, there are no previous reports about *MTRNR2L12* expression in surgical samples from patients with FCD. However, other authors have addressed studies on *MTRNR2* (also known as humanin) and demonstrated that the expression of the corresponding protein in human neurons (29) was associated with protective effects, such as anti-apoptotic by inhibiting BAX translocation from the cytosol to mitochondria (30), antioxidant by increasing superoxide dismutase activity (31), and preservation of synaptic connectivity by preventing dendritic atrophy caused by glutamate (32). Thus, should *MTRNR2L12* exert similar functions to those described for *MTRNR2*, it would be possible to consider a neuroprotective role for the presently detected transcript in an abnormal cortical circuitry with dysmorphic neurons, no balloon cells and chronically exposed to seizures.

Similar to what was performed for the cholesterol biosynthesis enzymes, we attempted to immunohistochemically validate our transcriptome result regarding the upregulation of *MTRNR2L12* in the gray matter of FCD IIa compared to IIb lesions. However, we found no commercial antibody available for any other validation method. Moreover, we customized a primary antibody against the protein presumably encoded by such transcript based on the high-throughput RNA sequencing data. Even though such antibody was tested both in FFPE and in frozen FCD samples, no conclusive cellular expression of the putative protein was detected. Thus, the tissue distribution of the protein encoded by *MTRNR2L12* and its role as a diagnostic neuropathological marker of FCD type IIa remain to be elucidated.

The analysis of the white matter transcriptome of FCD type II lesions yielded no results that we could straightforwardly associate with FCD or other brain malformations causing epilepsy. However, a review of each of the differentially expressed genes showed that *GPNMB* expression was significantly higher in the FCD IIb group than in FCD IIa and controls. Notably, such gene encodes the glycoprotein non-metastatic melanoma protein B (GPNMB), a molecule that plays a role in motility processes, such as invasion and metastasis, in poorly metastatic melanoma cells (33). Therefore, we hypothesized that this protein could be relevant to the pathophysiology of FCD, a malformation in which abnormal cell migration is also involved. Interestingly, the additional immunohistochemical investigation of GPNMB in the current brain samples showed positivity virtually only in balloon cells. Potential confounders, such as normal and abnormal neocortical neurons, as well as heterotopic neurons in the U-fibers, were not stained considering immunophenotypic and morphological features (34). Specifically, we recognized balloon cells by considering large and frequently rounded cells with abundant opaque cytoplasm, occasional multinucleation and immunopositivity for vimentin (10, 12, 16).

To our knowledge, there are no previous reports on GPNMB expression in FCD surgical samples or experimental models. Thus, a relevant finding of our study is that GPNMB may be a diagnostic neuropathological marker of balloon cells. In fact, we did not detect other cell types immunopositive for this molecule by using the primary antibody, whose clone is SP299. Such finding may help to overcome potential problems reported by other authors after using different markers to identify balloon cells. In fact, Yasin et al. (35) detected immunopositivity for β1 integrin not only in balloon cells, but also in smooth muscle, meninges and endothelium. Besides, GPNMB could be helpful to better characterize small surgical samples from individuals with clinical neuroimaging diagnosis of FCD type IIb but whose H&E-stained sections only show dysmorphic neurons or do not allow the distinction between balloon cells and reactive astrocytes. Particularly, balloon cells may express immunohistochemical astrocytic markers, such as vimentin and glial fibrillary acidic protein (GFAP) (35–39), thus hampering the unequivocal identification of these two cell types even with additional diagnostic techniques. In this context, the addition of anti-GPNMB antibody to the panel of immunohistochemical stainings currently recommended by the ILAE (10, 12, 16) could help with the histopathological identification of balloon cells and the precise classification of the dysplastic cortex. Future studies with larger cohorts from other centers are necessary to validate such finding and support its use in the diagnostic routine.

GPNMB has been described to have neuroprotective, reparative, or anti-inflammatory actions in neurodegenerative disorders (40–42) and cerebral ischemia-reperfusion injury (43). Under these circumstances, such protein was found to be synthesized by neurons, astrocytes, and microglia in both human and murine tissues. However, the corresponding pathophysiology mechanisms are still debatable. Conversely, in our study GPNMB was only observed in balloon cells. Such discrepancy with the mentioned reports could be due to technical matters, including primary antibody clonality. Irrespective of tissue distribution differences between our study and those from others, it is conceivable that GPNMB expression by balloon cells might be associated with neuroprotection, cellular reparation, and anti-inflammatory action. As proposed above, FCD type II may involve abnormalities in neurotransmission and maintenance of neuronal membrane structures as well as tissue inflammation (27, 28).

The restricted sample size is a limitation of our study. However, it is noteworthy that the number of individuals with FCD is larger than in previous reports (14, 15). In addition, despite the reduced sample sizes, we were able to validate the results regarding cholesterol and GPNMB at the protein level, which gives biological consistency to our findings.

In conclusion, our study provides novel and high-quality data about gene expression and enriched cellular pathways obtained from surgical samples of patients with FCD type IIa and IIb. The current genetic and histopathological results, suggesting cellular events associated with neuroprotection, may base future clinical and experimental approaches to further understand the pathophysiological events associated with FCDs and tissue response to seizures. Furthermore, upregulations of *MTRNR2L12* in FCD IIa and GPNMB in FCD IIb might be potential neuropathological biomarkers of a brain cortex chronically exposed to seizures and for balloon cells, respectively. Our results highlighted potential pathophysiological mechanisms and contributed to tissue characterization of a frequent cause of focal epilepsy.

## Data availability statement

The data presented in the study are deposited in the GEO repository, accession number GSE213488.

## Ethics statement

The studies involving human participants were reviewed and approved by the University of Campinas (UNICAMP; Campinas, Brazil) and the FAU/University Hospital Erlangen (Erlangen, Germany) Institutional Ethical Committees (UNICAMP—CEP#470/2003 and CAAE: 12112913.3.0000.5404; FAU—FP7 health program, DESIRE grant agreement #602531). Written informed consent to participate in this study was provided by the participants' legal guardian/next of kin.

## Author contributions

FR, IL-C, FC, AV, and IB contributed to the study design. GA-M, MCPA, JT, AS, GZ, PA, EG, HT, MKMA, VA, WS, CY, RC, IB, AV, FC, IL-C, and FR obtained and analyzed the data. GA-M, JT, MCPA, AV, IB, FC, IL-C, and FR drafted the manuscript and figures. All authors reviewed and approved the final version of the manuscript.

## Supplementary material

Supplementary material Transcriptome data (enriched pathways and gene expression) of focal cortical dysplasia (FCD) ILAE type IIa (FCD IIa) and FCD ILAE type IIb (FCD IIb) samples. Data of the gray and white matters are presented separately. Datasheets 1 and 2 show the enriched pathways, *p*-values and corresponding differentially expressed genes of the gray matter of FCD IIa and IIb groups, respectively, vs. controls (autopsy). Datasheets 3–8 present gene symbols (or Ensembl code when gene symbol was not available), Fold Change (log_2_fold change) values relative to the groups indicated in each datasheet designation, *p*-values and adjusted *p*-values. Ctrl, control; GM, gray matter (cortical layer); WM, white matter.
